# A Novel Chaotic Map and an Improved Chaos-Based Image Encryption Scheme

**DOI:** 10.1155/2014/713541

**Published:** 2014-07-20

**Authors:** Xianhan Zhang, Yang Cao

**Affiliations:** ^1^Department of Mathematics, Southeast University, Nanjing 210096, China; ^2^Department of Mechanical Engineering, The University of Hong Kong, Pokfulam, Hong Kong; ^3^College of Telecommunications and Information Engineering, Nanjing University of Posts and Telecommunications, Nanjing 210003, China

## Abstract

In this paper, we present a novel approach to create the new chaotic map and propose an improved image encryption scheme based on it. Compared with traditional classic one-dimensional chaotic maps like Logistic Map and Tent Map, this newly created chaotic map demonstrates many better chaotic properties for encryption, implied by a much larger maximal Lyapunov exponent. Furthermore, the new chaotic map and Arnold's Cat Map based image encryption method is designed and proved to be of solid robustness. The simulation results and security analysis indicate that such method not only can meet the requirement of imagine encryption, but also can result in a preferable effectiveness and security, which is usable for general applications.

## 1. Introduction

With the discovery of a series of chaotic maps such as Tent Map [1, 2] and Logistic Map [3], researchers and scholars have been able to apply them into a variety of fields. The knowledge of chaotic maps is perhaps one of the most significant achievements in nonlinear science. Since 1980s, researches on chaos theory have been overlapping and mixing up with other subjects, in the meanwhile promoting their further developments. The fields that take advantage of knowledge concerning chaos range greatly from math and astronomy to music and art. Besides, the most famous magazines in the world such as Nature and Scientific American once published a great deal of discoveries and progresses in chaos theory [4]. Therefore, it is reasonable to judge that chaos has been becoming a universal language between these important subjects.

If we are to further classify the applications of the chaos in different categories, chaos analysis [5] and chaos synthesis [6] will be the answer. As for the former, based on complex manual work and natural system, we tend to find some hidden rules inside of them. One example is the prediction towards time series [7–10]. For the latter, by using manually produced chaotic system, we are inclined to discover some possible functions contained within the chaotic dynamics [11–13].

In addition, some likely applications of the chaos are listed below. First, combining neural network and chaos, we utilize chaotic status of intermediate processes to let networks avoid the partial minimum point. And hence it guarantees global optimum according to [14]. Second, the chaos theory has already been used in high-speed searching process. Last but not least, chaotic maps are widely applied in secure communication which is carefully studied in [13, 15]. We could not only use chaotic signals to encrypt the information needed to be secure but also decipher encrypted one as well according to [16–18]. Also, researches regarding these aspects are known to have already been put in the national defense plan of China.

Despite the fact that the fields that call for chaotic maps range greatly, one thing they share in common is that they all need the chaotic features of chaotic maps. In other words, the feature that a simple initial point and a given value of the parameter could completely control the whole process is what we need. As a matter of fact, chaotic maps are quite sensitive to the initial point, which means even a very slight change in the value of initial point would result in a dramatic change of the sequence produced by the chaotic map. However, at present, only a limited number of one-dimensional chaotic maps (e.g., Tent Map and Logistic Map) are introduced. Also, their properties are somehow limited and may no longer satisfy our needs. Too often our methods of encryption and engineering projects are merely based on these simple chaotic maps. Without new and better chaotic maps, our applications will remain unchanged and might get stuck in the future. This may lead to an urgent need for more and better chaotic maps.

In this paper, a new one-dimensional chaotic map is first introduced, and we use the maximal Lyapunov exponent [19–21] to determine how well the map performs. In addition, we later prove that this new chaotic map actually exhibits a larger maximal Lyapunov exponent, indicating better properties of the chaotic map. What is more is that a new algorithm based on this new chaotic map is used in image encryption, providing a brand new way to encrypt images. Compared with previous ways to encrypt image, it not only utilizes the excellent chaotic property of the newly discovered map itself but also entails another classical map: Arnold's Cat Map [22–24], through which coordinates of the target image's grey value matrix will be changed to another. Lastly, a security analysis is accomplished by plotting histogram of the image's grey values and calculating information entropy [25]. Without any knowledge as to how many times the target image is iterated, it is next to impossible to decrypt the encrypted image. Therefore, the safety of the image is largely strengthened and guaranteed. Now we discuss this new chaotic map and how to use it to encrypt images in detail in the following.

## 2. Design and Analysis to the New Chaotic Map

In this section, we first discuss the definition of “maximal Lyapunov exponent.” Then, we plot the Lyapunov spectrum of the two traditional one-dimensional chaotic maps. Next, a new chaotic map is introduced. Lastly, a comparison between the new chaotic map and two traditional maps is carefully made.

### 2.1. Maximal Lyapunov Exponent

#### 2.1.1. Definition of the Lyapunov Exponent

According to statements in [19, 26], Lyapunov exponent *λ* usually represents the features of a chaotic system, named after the great Russian mathematician Lyapunov.

For discrete system (maps or fixed point iterations) *x*_*n*_ = *f*(*x*_*n*−1_) and for an orbit starting with *x*_0_, the Lyapunov exponent can be defined as follows:

(1) $$\begin{array}{c} \lambda \left(x_{0}\right)=\underset{n\to \infty }{\lim}\frac{1}{n}\overset{\infty }{\underset{i=1}{\sum }}\ln\left|f^{\prime }\left(x_{i}\right)\right|. \end{array}$$

It is common to refer to the largest *λ* defined by (1) as the maximal Lyapunov exponent because it determines a notion of predictability for a chaotic system.

#### 2.1.2. Properties of Maximal Lyapunov Exponent (MLE)

A positive *λ* is usually taken as an indication that the system is basically chaotic. Besides, it is also apparently true that the larger MLE is, the more chaotic the map is. And this means a better chaotic map according to [20].

**Remark 1 .**

Lyapunov exponent, as an important exponent to test the property of chaotic map, is widely used in the world of chaos. Actually, from the definition equation (1), we could clearly find out that Lyapunov is an average value of ln⁡|*f*′(*x*)|. Since |*f*′(*x*)| is the indicating parameter that measures the variation speed for *f*(*x*), Lyapunov exponent, the average value of ln⁡|*f*′(*x*)| is bound to reflect the chaotic properties of *f*(*x*). Thus, maximal Lyapunov exponent can largely expresses the overall performance of chaotic maps.

Next, we begin with calculating the MLE and plotting the Lyapunov exponent spectrum for two typical one-dimensional maps, Tent Map and Logistic Map.

### 2.2. Tent Map

In mathematics, according to [1], Tent Map with parameter *μ* is a real-valued function *f*(*μ*) defined by

(2) $$\begin{array}{c} f\left(\mu \right)=\mu \;\min\left\{x,1-x\right\};\;\mu \in \left(0,2\right). \end{array}$$

**Figure 1:**
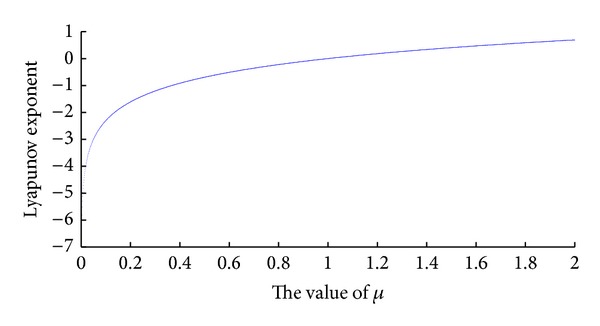
Lyapunov exponent spectrum of Tent Map.

Thus, we can obtain the Lyapunov exponent spectrum of Tent Map, which is shown in Figure 1.

In addition, we could calculate the MLE for Tent Map, which is 0.6931 (when *μ* → 2), indicating that the map itself is chaotic.

### 2.3. Logistic Map

Logistic Map [27, 28] is a polynomial mapping of degree 2 that exhibits chaotic behavior. The Logistic Map equation is given by

(3) $$\begin{array}{c} x_{n+1}=\mu x_{n}\left(1-x_{n}\right). \end{array}$$

**Figure 2:**
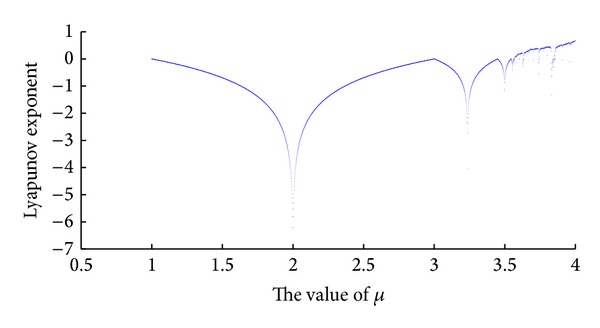
Lyapunov exponent spectrum of Logistic Map.

When the variable *μ* is given different value, ranging from 2 to 4, through formula (1), we could plot Lyapunov exponent of Logistic Map as well. That is shown in Figure 2.

It is obviously shown in Figure 2 that, when *μ* → 4, MLE of Tent Map is reached. Through calculating, maximal Lyapunov exponent of the Logistic Map is 0.6785.

### 2.4. A New Chaotic Map

When we replace “*x*” with “1 − 2|*x*|” in Logistic Map, formula (4) will be attained:

(4) xn+1=2μ|xn|(1−2|xn|); (−1<xn<1).

**Figure 3:**
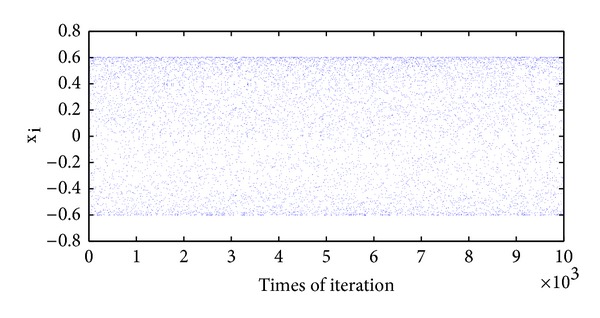
Scattered 10000 points gained from the new chaotic map.

If we choose *x*_0_ = 0.4 as the initial point of the map and the parameter *μ* = 2.4140, after 10000 times of iterations, we will get the randomly scattered image of the map in Figure 3 when we plot every *x*_*i*_  (*i* = 1,2,…).

**Remark 2 .**

It is clearly displayed in Figure 3 that the sequence generated from the new chaotic map ranges from −0.6 to 0.6, while, in Tent Map and Logistic map, it is a little larger, ranging from −1 to 1. However, this does not matter that much, since we consider the range that they share in common, which is −0.6 < *x*_*n*_ < 0.6.

**Figure 4:**
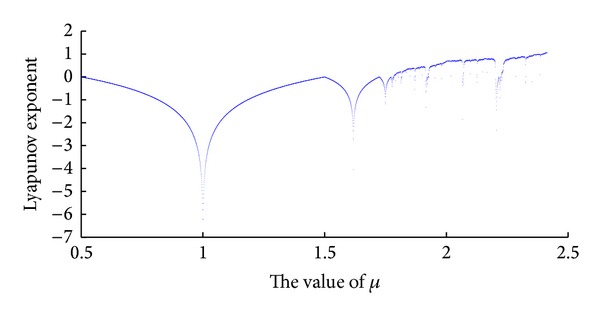
Lyapunov exponent spectrum of the new map.

In order to see if the map is a chaotic map and, if yes, how good the map is, similarly, we also use the maximal Lyapunov exponent to see that, starting with *x*_0_ = 0.4 and iterating 2000 times. Hence, Figure 4 will be yielded.

As is shown in Figure 4, when *μ* = 2.4140, the MLE of the new chaotic map reaches beyond 1, to be exact, 1.0742.

**Figure 5:**
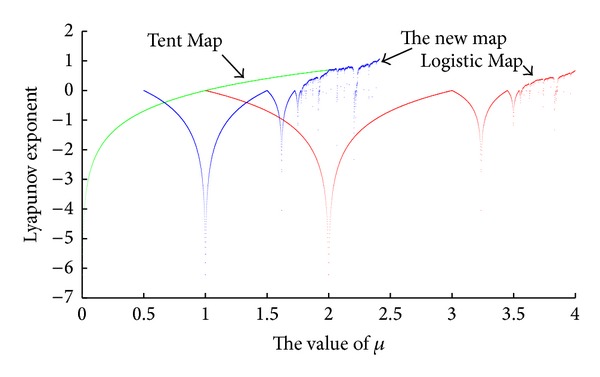
Lyapunov exponent spectrum comparison with three chaotic maps.

Next, we combine the three Lyapunov exponent spectrums given above together and Figure 5 is the result.

**Remark 3 .**

Just like what we have discussed in Section 2.1, a larger maximal Lyapunov exponent indicates not only a stronger sensitivity to the initial point but also it also indicates that the chaotic system itself is “more chaotic.” In other words, the chaotic map with larger MLE is of better quality. It is demonstrated above that the MLE of the two most typical chaotic maps (2) and (3) all end below 1, which are apparently smaller than the new map (4). The MLE of the new chaotic map, as expected, has reached beyond 1. Therefore, the new map (4) is supposed to bear the potential to perform more effectively in engineering or encryption process than the two classic ones (2) and (3) mentioned above.

## 3. Application on Image Encryption

### 3.1. Encryption Scheme

In this section, we are about to take one step further, applying the new map on image encryption.

Now, we begin introducing an image encryption algorithm based on the map we have just constructed.

*Step 1. *For given initial point *x*_0_ and the parameter *μ*, we could simply use the new map (4) to produce a sequence *m*_*k*_ ∈ (−0.6, 0.6)  (*k* = 1,2,…). Consider

(5) mn+1=2μ|mn|(1−2|mn|) (−0.6<mn<0.6).

Then, in order to make |*x*_*k*_| reach closely 1, we choose *x*_*k*_ = (5/3)*m*_*k*_; (*k* = 1,2,…).

*Step 2. *Next, we transform decimal numbers *x*_*k*_ to the form of binary numbers, and consequently we will get *x*_*k*_′. After that, we choose the first 8 figures after the decimal point of *x*_*k*_′ to form a new binary number, *B*_*k*_.

To put in the language of math, that is:

(6) $$\begin{array}{c} x_{k}^{\prime }=\overset{\infty }{\underset{v=0}{\sum }}a_{k,v}2^{-(v+1)};\;a_{k,v}=0\;\text{or}\;1.\\ x_{k}^{\prime }=\overset{7}{\underset{v=0}{\sum }}a_{k,v}2^{-(v+1)}=2^{-8}\overset{7}{\underset{0}{\sum }}a_{k,v}2^{7-v}=2^{-8}B_{k;}\\ B_{k}=a_{k,v}2^{7-v}. \end{array}$$

Thus, for each *x*_*k*_, there is a unique *B*_*k*_ corresponding to it. Obviously, *B*_*k*_ is formed by the first 8 numbers after the decimal point of *x*_*k*_′. In this way, we could obtain the original chaotic sequence in the form of binary numbers with 8 places. Similar approaches have been discussed in [29].

**Remark 4 .**

Binary numbers with 8 places have three major advantages that are listed below. First of all, it corresponds well with (*R*, *G*, *B*), which is the gray values' row vector of each point in an image. *R* refers to red, *G* refers to green, and *B* refers to blue. In fact, they all range from 0 to 255. And, hence, if they are written into binary forms, 8-place binary numbers will be exactly what we get. Therefore this step provides convenience to the following steps. Secondly, computers have always been using binary numbers to operate. Thus binary numbers tend to make calculation efficient and time-saving. Thirdly, as per what we have discussed in Section 2.1, the sequence generated by the chaotic map is basically random and therefore this will lead the binary numbers to be random as well due to the transmission of randomness.

*Step 3. *Suppose that the pixel of the target image is *m* × *n*, and then we put *B*_*k*_ produced in Step 1 into a matrix AA with the size *m* × *n*. In other words, from left to right and from up to down, each *B*_*k*_ is assigned to a unique, particular position in matrix AA.

*Step 4. *For point (*i*, *j*) from the plaintext image we have its grey value vector (*R*, *G*, *B*) written in binary form. In order to build a connection between the grey value matrix of the plaintext image and the chaotic sequence matrix AA, it is reasonable to think of the XOR operation (⊕), the definition of which is given below in accordance with [30]:

(7) 1⊕0=1;  1⊕1=0.0⊕0=0;  0⊕1=1.

Thus, we use XOR to “make a mess.” For point (*i*, *j*) from the plaintext image, we also extract AA(*i*, *j*) from AA, which is made up with the chaotic sequence generated. Next, we do XOR operation as follows:

(8) $$\begin{array}{c} R_{1}=R⊕\text{AA}\left(i,j\right).\\ G_{1}=G⊕\text{AA}\left(i,j\right).\\ B_{1}=B⊕\text{AA}\left(i,j\right). \end{array}$$

*Step 5. *In Step 6, we make a change to the coordinate of (*i*, *j*). To be exact, let $\left(\begin{array}{c} i\\ j \end{array}\right)$ be the starting column vector, which is $\left(\begin{array}{c} X_{{\;}_{0}}\\ Y_{0} \end{array}\right)$, and then iterate a given “*k*” times with formula (9) according to [23, 24]

(9) $$\begin{array}{c} \left(\begin{array}{c} X_{n+1}\\ Y_{n+1} \end{array}\right)=\left(\begin{array}{cc} a & b\\ c & d \end{array}\right)\left(\begin{array}{c} X_{n}\\ Y_{n} \end{array}\right)\mathrm{mod}m,\;\left(ad-bc=1\right).\;\;\;\;\; \end{array}$$

**Figure 6:**
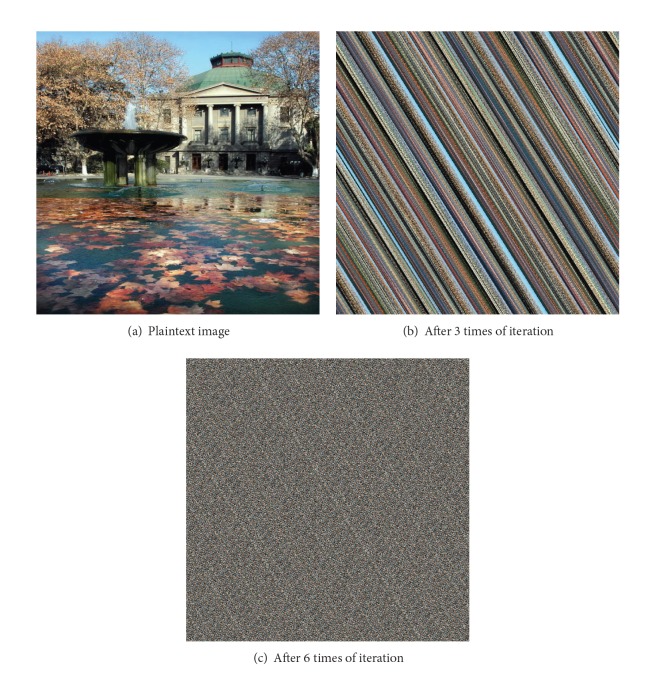
Sample experiment based on Arnold's Cat Map.

After iteration of a given “*k*” times, we will obtain a new coordinate for point (*i*, *j*) in the plaintext image, and we mark it as (*i*′, *j*′). This is one of the most famous coordinate change maps, the Arnold's Cat Map. Lastly, we assign the value of (*R*_1_, *G*_1_, *B*_1_) from (*i*, *j*) to (*i*′, *j*′). (Tips: “mod” in the formula means complementation, if the plaintext image is of the size *x*_*a*_ × *y*_*a*_ (the pixel value); *m* = max⁡{*x*_*a*_, *y*_*a*_}.) A sample experiment has been provided in Figure 6.

**Remark 5 .**

There exists actually a slight defect of Step 5, due to the fact that, after a special number of iteration times, the image could simply be restored as in the beginning according to [22]. As Arnold's Cat Map is defined, it is a periodic map, and this property leads to an unsafe encryption. Therefore, it is very significant to choose a proper value of “*k*,” the number of times of iterations to prevent the image from being restored.

**Remark 6 .**

Along with defects, there are also huge advantages. First, only a few times of iteration are enough to guarantee the thorough change of the picture. Without knowing the times of iteration, it takes next to forever to decipher the image, as you could see as follows. Second, “mod” guarantees that the size of the image will be exactly the same if the picture is square-sized. Lastly, there is no denial that it is easy to recover the original image using some fundamental knowledge from algebra. Elaborate discussion will be in Section 3.2.

*Step 6. *Repeat Steps 3–5 for every point in the target plaintext image, and use these processed grey values to form an encrypted image.

### 3.2. Decryption Scheme

*Step 1. *Read the encrypted image, and then write its grey value into a three-dimensional matrix.

*Step 2. *According to Step 6 in encryption, we could simply do the opposite: start with (*i*′, *j*′) in the encrypted image, use the formula listed below, and iterate “*k*” times as well:

(10) (Xn+1Yn+1)=1ad−bc(d−b−ca)(XnYn)mod⁡m(ad−bc=1).

The definition of “*m*” is the same as the one mentioned above, and thus we attain the original coordinate of (*i*, *j*).

*Step 3. *See Steps 1 and 2 in the encryption part.

*Step 4. *In order to restore changes from Step 5, just do XOR operation with (*R*_1_, *G*_1_, *B*_1_) in points (*i*, *j*) and AA(*i*, *j*) again. Hence, (*R*, *G*, *B*) is obtained. That is,

(11) $$\begin{array}{c} R=R_{1}⊕\text{AA}\left(i,j\right).\\ G=G_{1}⊕\text{AA}\left(i,j\right).\\ B=B_{1}⊕\text{AA}\left(i,j\right). \end{array}$$

*Step 5. *Use (*R*, *G*, *B*) of every point to form the original image. Hence, restoration of the plaintext image is accomplished.

### 3.3. Overall Process of Image Encryption

**Figure 7:**
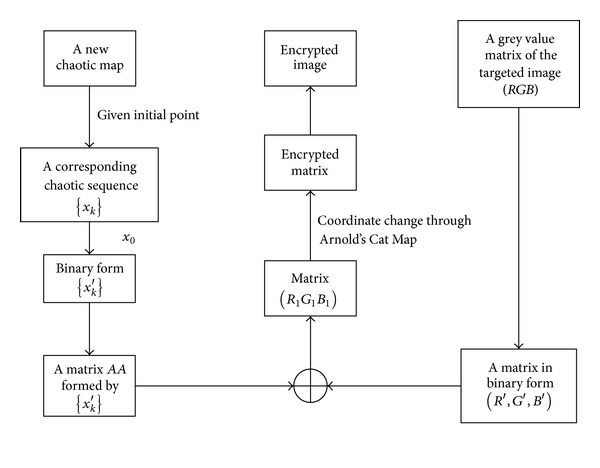
Proposed encryption scheme.

Statements mentioned above yield the encryption scheme listed in Figure 7.

### 3.4. Simulation Results

**Figure 8:**
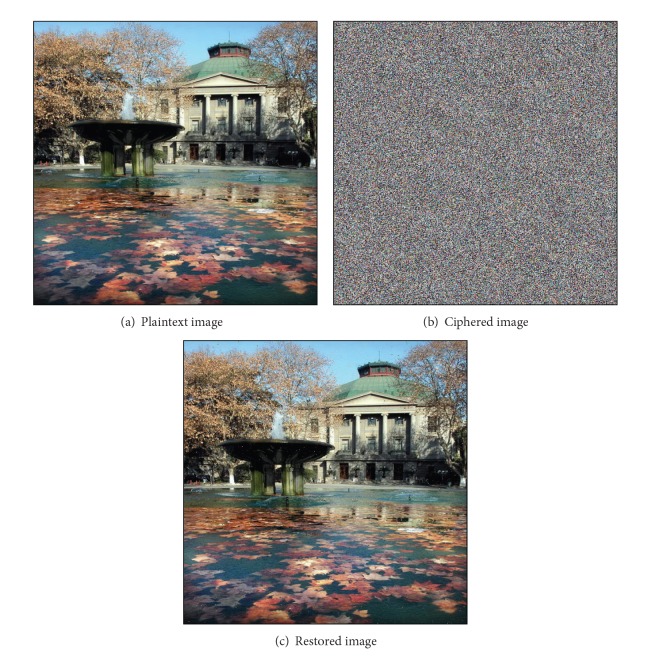
Simulation results.

Take the image of Figure 8(a) as a sample to test the algorithm mentioned above and the plaintext image, encrypted image, and restored image are given in Figure 8.

## 4. Security Analysis

In this section, by plotting the histogram and calculating the so-called “information entropy,” we test the security qualities of the proposed method in Section 2.

### 4.1. Histogram Analysis

**Figure 9:**
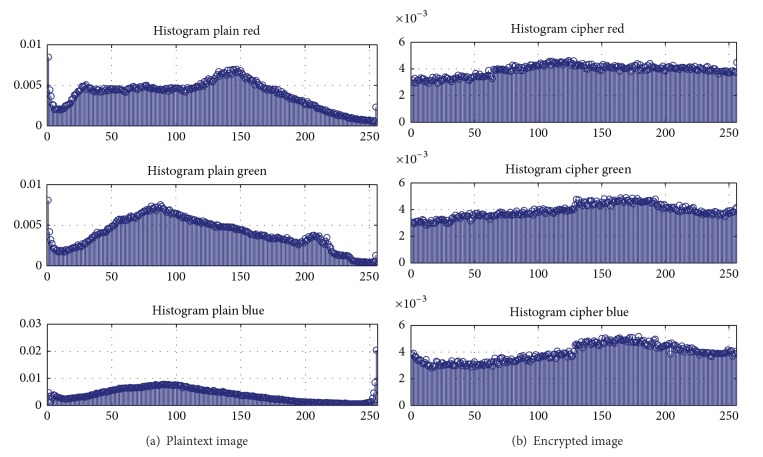
Histogram analysis results.

There is no denying that a well-ciphered image should provide no chances for the attackers to decrypt through statistics analysis. On the one hand, the grey level of the plaintext image in Figure 8(a) is somehow similar to normal distribution in accordance with Figure 9(a) and this would bring about vulnerabilities to decryption. On the other hand, the ciphered image in Figure 8(b), though not perfectly average on each value between 1 and 256, is somehow subjected to uniform distribution according to Figure 9(b), which undoubtedly adds up difficulty for the attacker to decrypt according to [16].

### 4.2. Information Entropy

According to the thesis in [25], the value of information entropy typically expresses the feature of randomness. Its definition is given by

(12) $$\begin{array}{c} H\left(m\right)=-\sum p\left(m_{i}\right)\text{lo}{\text{g}}_{2}\frac{1}{p\left(m_{i}\right)}, \end{array}$$

where “*m*” refers to message and *p*(*m*_*i*_) represents the chances of appearance of the *i*th message. As for images, *p*(*m*_*i*_) stands for the probability of a particular grey value (*R*, *G*, *B*). Under this circumstance, *H*(*m*) is also called “image entropy.” The entropy of a perfectly encrypted image should, in ideal case, approach 24, since 3 × log_2_256 = 24 (3 means 3 color planes) according to [14, 31]. The entropy *H*(*m*) = 23.9616 is yielded through calculating, which is extremely close to 24. Therefore it can be inferred that the encrypted image is almost random. In this way, we tend to believe that the safety of the image is largely promoted and ensured.

**Remark 7 .**

As the result of the entropy shows, *H*(*m*) is perfectly close to 24, which is an ideal entropy for a randomly encrypted image. And this result in turn proves the excellent chaotic properties of the new chaotic map. What is more is that the comparison between the histograms of the plaintext image and the encrypted image shows that the new chaotic map actually makes grey values distribute uniformly, which convinces us of the fact the new chaotic map is of robust quality.

## 5. Conclusion

In this paper, a new chaotic map with better chaotic properties has been proposed. To step further, a comparison with traditional one-dimensional chaotic maps has been made as well through their maximal Lyapunov exponent spectrums, which proves the new chaotic map's marvelous application prospect. In addition, it has been applied on image encryption. Along with Arnold's Cat Map, it could successfully produce a ciphered image based on original plaintext image. Steps and the proposed scheme to encrypt a targeted image and decrypt a ciphered image have been given. What is more is that the results of encryption and decryption simulation have been provided. At last, security reliability towards this algorithm has been discussed. As a result, this method is of excellent quality and robustness and turns to be theoretical unbreakable by convention attacks without any knowledge to the values of the starting point and parameters. Yet, to seek perfectness, a safe conveyance of the parameter still remains to be a problem. That is, when the attacker gains the parameter value and initial point value, a whole system can break down easily. Thus a safe transmission of keys may be our next direction to study.
